# A Deep Learning approach for Diagnosis of Mild Cognitive Impairment Based on MRI Images

**DOI:** 10.3390/brainsci9090217

**Published:** 2019-08-28

**Authors:** Hamed Taheri Gorji, Naima Kaabouch

**Affiliations:** Department of Electrical Engineering, University of North Dakota, Grand Forks, ND 58202-7165, USA

**Keywords:** deep learning, convolutional neural network, mild cognitive impairment, Alzheimer’s disease

## Abstract

Mild cognitive impairment (MCI) is an intermediary stage condition between healthy people and Alzheimer’s disease (AD) patients and other dementias. AD is a progressive and irreversible neurodegenerative disorder, which is a significant threat to people, age 65 and older. Although MCI does not always lead to AD, an early diagnosis at the stage of MCI can be very helpful in identifying people who are at risk of AD. Moreover, the early diagnosis of MCI can lead to more effective treatment, or at least, significantly delay the disease’s progress, and can lead to social and financial benefits. Magnetic resonance imaging (MRI), which has become a significant tool for the diagnosis of MCI and AD, can provide neuropsychological data for analyzing the variance in brain structure and function. MCI is divided into early and late MCI (EMCI and LMCI) and sadly, there is no clear differentiation between the brain structure of healthy people and MCI patients, especially in the EMCI stage. This paper aims to use a deep learning approach, which is one of the most powerful branches of machine learning, to discriminate between healthy people and the two types of MCI groups based on MRI results. The convolutional neural network (CNN) with an efficient architecture was used to extract high-quality features from MRIs to classify people into healthy, EMCI, or LMCI groups. The MRIs of 600 individuals used in this study included 200 control normal (CN) people, 200 EMCI patients, and 200 LMCI patients. This study randomly selected 70 percent of the data to train our model and 30 percent for the test set. The results showed the best overall classification between CN and LMCI groups in the sagittal view with an accuracy of 94.54 percent. In addition, 93.96 percent and 93.00 percent accuracy were reached for the pairs of EMCI/LMCI and CN/EMCI, respectively.

## 1. Introduction

Mild cognitive impairment (MCI), which is considered as a potential forerunner to Alzheimer’s disease (AD) and other types of dementia, is a condition in which individuals have a slight but considerable and measurable decline in their mental and cognitive abilities. Although MCI does not always lead to dementia, people with MCI, mainly those who complain about memory problems, are more likely to develop AD [1,2]. The four-year conversion rate between all MCI subtypes to dementia and AD is 56 percent, and 46 percent, respectively [3], and the probability of conversion from MCI to AD is almost seven times higher than in other elderly people [4]. Therefore, MCI can be considered as a transitional state between being healthy and having Alzheimer’s [5].

Currently, AD is the sixth leading cause of death in the United States [6]. Approximately 5.7 million Americans are living with AD [7] and this number is predicted to increase to 13.8 million in 2050 [8]. This rapid growth in AD, not only affect the patients and their families, but it is also one of the challenges facing governments. In 2019, AD and other types of dementia have been estimated to cost the U.S. approximately $290 billion, and based on this estimation, the cost is expected to increase to more than $1.1 trillion by 2050 [9].

The ability to accurately and reliably diagnose MCI can lead to benefits to both individuals and governments. The persons diagnosed as having MCI are able to undergo additional technologically advanced and precise tests to find out their current stage, and if it is due to AD or not. With the diagnosis of MCI due to AD, they can be eligible for a number of clinical trials. Moreover, individuals and their families can have more time to plan for social, financial, and medical decisions. Further, it can lead to considerable savings in medical and long-term care costs for both the patients and governments. For example, in the U.S., if all the AD patients were diagnosed in the early stages (during MCI stage), it would save a total of $7 trillion to $7.9 trillion [7].

In recent years, based on the new criteria defined by the Alzheimer’s Disease Neuroimaging Initiative (ADNI) (ADNI go, ADNI 2), MCI patients have been classified into two groups: Early MCI (EMCI) and late MCI (LMCI). These two groups are discriminated from each other based on the degree of memory impairment. In the EMCI patients, the decline in memory is approximately between 1.0–1.5 standard deviations (SD) below the normative mean, while in LMCI, the decline in memory is at least approximately 1.5 SD below the normative mean [10]. Due to the similarities between the normal aging and MCI patients’ brain structures, a diagnosis of the MCI stage based on MRI and the discrimination between these two groups, mainly between EMCI and normal aging, is one of the most challenging parts of aging research.

Brain imaging methods, such as magnetic resonance imaging (MRI), functional magnetic resonance imaging (fMRI), positron emission tomography (PET), single-photon emission computed tomography (SPECT), and computed tomography (CT) are prevalent methods researchers and doctors use to diagnose MCI and AD in patients [11,12].

There are many features in brain images which can be used for a diagnosis and to discriminate between MCI, AD, and healthy people. The cerebral atrophy rate is one of the features that is significantly greater in MCI and AD patients than in normally aging people [13,14]. Ventricle enlargement [15], hippocampal atrophy, and the rate of change of brain atrophy are the other features that are usually used by scientists [16,17]. The changes in gray matter (GM), white matter (WM) and cerebrospinal fluid (CSF) are three other main features of MCI and AD patients [18,19,20]. Given that, the WM and CSF are considered as the two main features for the diagnosis of MCI, and researchers show that the GM atrophy is more associated with the cognitive performance in MCIs [21,22,23].

The statistical parametric method (SPM) opened a new window for measuring the spatial distribution of atrophy in brain aging research [24,25,26]. The SPM was created by Karl Friston and uses statistical techniques for analyzing brain activity in MRI, fMRI, or positron emission tomography (PET) scans [27].

Due to the engineering field of science, it was possible to create computer-aided diagnosis (CAD) systems, which play a crucial role in assisting the researchers and doctors in their interpretation of medical imaging. In recent years, the use of the machine learning approach, especially deep learning methods in CAD systems for the diagnosis and classification of healthy control normal (CN) people, MCI and AD patients, has exponentially increased [28,29,30]. Gorji et al. developed a Zernike moment-based method for the early diagnosis and classification between AD, MCI and normal control groups from structural MRIs, achieving an accuracy rate of 97.27%, 95.59% and 94.88% for AD/CN, MCI/CN, and AD/MCI respectively. They proposed a novel method that uses Pseudo Zernike moments (PZMs) to extract discriminative information from the MR images of the three mentioned groups. Furthermore, they used two types of artificial neural networks (pattern recognition and learning vector quantization (LVQ) networks), to classify the extracted information of the MRIs.

Ramirez et al. [31] proposed a CAD system for the early detection of AD based on a partial least squares (PLS) regression model and a random forest (RF) predictor using SPECT. They found that PLS outperformed PCA as a feature extraction method with the highest sensitivity, specificity, and accuracy values of 100%, 92.7%, and 96.9%, respectively. Furthermore, their proposed PLS-RF method outperformed other CAD systems, such as a principal component analysis (PCA)-RF, Gaussian mixture models (GMM)-support vector machine (SVM) and voxel-as-features (VAF)-SVM methods.

In deep learning, convolutional neural networks (CNNs) are widely known for their abilities for image recognition, segmentation, and classification [32,33,34]. One of the main advantages of CNNs is that, unlike the conventional machine learning methods, there is no need for a manual feature extraction step, and CNNs are able to extract the efficient features automatically and then classify the images.

Current research is directed towards the development of a CNN-based method for the diagnosis and classification of the CN, EMCI and LMCI groups based on GM images extracted from MRIs by SPM. This can be very challenging, but also helpful for the early diagnosis of AD and bring other benefits as mentioned earlier.

## 2. Materials

### 2.1. Database

The data used in this study were acquired from the Alzheimer’s disease Neuroimaging Initiative (ADNI) database (http://www.loni.ucla.edu/ADNI). The ADNI was funded in 2004 by the National Institute on Aging (NIA), the National Institute of Biomedical Imaging and Bioengineering (NIBIB) and a contribution from many pharmaceutical companies and foundations. The measurement of the progression of the early AD and MCI was the primary goal of ADNI, which was investigated by a combination of clinical and neuropsychological assessments, serial MRI, PET, and other biological markers.

In this study, MRIs of a total of 600 subjects were used, which included 200 EMCI, 200 LMCI patients, and 200 CN individuals. The demographic information of all subjects in the three groups of cognitively normal (CN), EMCI, or LMCI are shown in Table 1.

### 2.2. Convolutional Neural Network

The convolutional neural networks (CNNs) are a type of deep artificial neural network which was inspired by a hierarchical model of the visual cortex [35]. The main application of the CNNs are in image recognition and classification [33], video classification [36], and medical image and signal analyzing [37,38,39]. A CNN typically consists of one or more convolution layers, one or more optionally down-sampling layers, a non-linearity layer, and a fully connected layer.

In CNNs, for classifying the input images, each image passes through the convolutional layer. The convolutional layer is comprised of a collection of filters, which plays a crucial role in CNNs architecture. A series of learnable filters are applied to the input images and detect specific patterns and features. This set of filters slide over the input image by a certain amount called the stride value. The stride determines the number of pixels that the filter shifts across the input image matrix. By convoluting between the filters and each part of the input image, a dot product is computed to produce a set of 2D feature maps.

The non-linearity layer, which also is named the activation layer, is used to introduce non-linearity to the CNN because during the convolutional layer, only linear calculations are computed.

In recent years, rectified linear units (ReLU) [33] have been used more as a non-linear function than the other non-linear functions, like sigmoid or hyperbolic tangents, because by means of ReLU (f(x) = max (0, x)) a CNN can train much faster due to the computational efficiency without a notable penalty to the generalization accuracy [33]. Moreover, using ReLU can be very helpful to decrease the vanishing gradient [40].

The pooling layer is the next layer of a CNN and is usually positioned between the convolutional layers. The pooling layer performs down-sampling and reduces the spatial dimension of the feature maps. Therefore, it cannot only reduce the network computational complexity, but also control overfitting. The function of the pooling layers are like the convolutional layers, but instead of doing the convolution operation, it moves over the feature map and replaces the value of each region with its maximum or average, using the defined pool size and stride.

The fully connected (FC) layer, which is the other name for the conventional neural network, computes the class score to generate the outputs equal to the number of classes desired by the CNN. The term, fully connected, implies that all neurons in a fully connected layer are connected to all the neurons in the previous layer. In the end, a sigmoid or softmax classification layer can be used in accordance with the binary or multi-classification task, respectively.

## 3. Methodology

### 3.1. Image Preprocessing

In this study, as previously mentioned, the MRIs from the ADNI database have been used. All the data were downloaded with the Neuroimaging Informatics Technology Initiative (NIfTI) format. SPM12 has been used for preprocessing. This study focused on GM because atrophy in GM can be considered as a prominent early AD biomarker [41] and, as it is mentioned earlier, GM is more associated with the cognitive performance in MCIs.

For preprocessing the neuroimages, three main steps were applied to the data which are explained in the following:

#### 3.1.1. Segmentation

By means of SPM segmentation, the brain tissue can be automatically segmented into three main parts, which are GM, WM, and CSF. This paper set the bias regularization on the light regularization (0.001), the full width at half maximum (FWHM) of Gaussian smoothness of bias on the 60 mm cutoff, and affine regularization on the ICBM space template. Moreover, for the spatial normalization of the data to the Montreal Neurological Institute (MNI) spaces, the deformation field was set in the forwarding mode.

#### 3.1.2. Normalization

After the segmentation of the data, GM images were considered for further analysis because, as mentioned before, GM is a significant early AD biomarker. For normalizing all GM images to MNI space, this paper set the written normalized images voxel size on (2 2 2) mm and, for sampling the images to MNI space, the 4th-degree B-Spline for interpolation was considered.

#### 3.1.3. Smoothing

**Figure 1:**
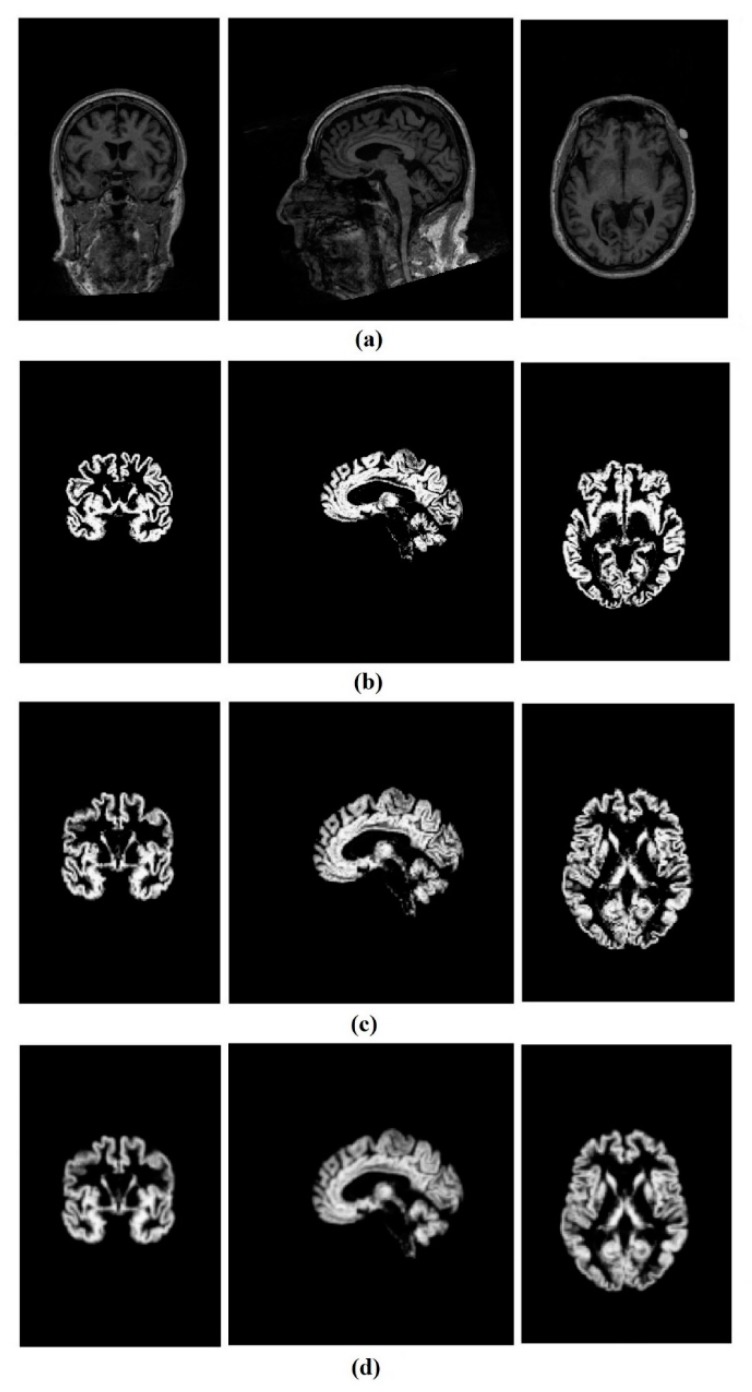
A control healthy subject’s MRI, (**a**) from left to right sagittal, coronal and axial view, (**b**). gray matter, (**c**). gray matter after normalization, (**d**). gray matter after smoothing.

Finally, all normalized GM images were smoothed by a Gaussian kernel. The FWHM of the Gaussian smoothing kernel were set on (2 2 2) mm. The results of the preprocessing steps are shown in Figure 1.

The original size of the data was 176 × 240 × 256, and after the preprocessing steps, all the GM images were reduced to the size of 79 × 95 × 79. For further analysis, 3D images were decomposed into 2D slices along the third direction, which named axial, coronal, and sagittal views. Then, all the 2D.nii GM files were converted to a portable network graphics (PNG) format and resized to 64 × 64 pixels by means of MATLAB (2018b) to be useable by our CNN. In each view of the MR images, there were some slices at the beginning and the end which did not contain any useful information, so they were removed, and consequently, 20 images of each view were considered for further processing (60 images per subject).

### 3.2. CNN architecture

**Figure 2:**
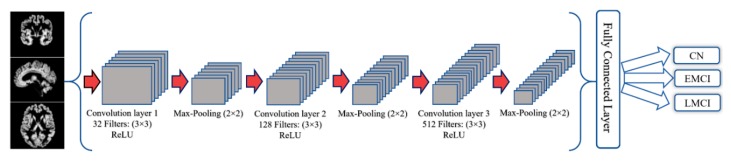
The architecture of the convolutional neural network.

The CNN architecture used in this study is composed of three convolutional layers which take an input image with a size of 64 × 64 (see Figure 2). All three convolutional layers were followed by a max-pooling layer. The 32 filters with a kernel size of 3 × 3 were considered for the first convolutional layer and the max-pooling layer kernel size was set on 2 × 2. The second and third layers comprised of 128 and 512 filters respectively, with the same filter and max-pooling kernel sizes as the first convolution layer. It is worthwhile to mention that ReLU was used as the activation functions in all convolutional layers. Moreover, the glorot uniform [42] kernel and the bias initializer were used for initializing the layer’s weights.

Finally, a fully connected layer with 128 input neurons and a ReLU activation function were used. Further, one output neuron followed by a sigmoid activation function was used in order to facilitate the binary classification. To prevent over-fitting, kernel and bias regularizers and also, the dropout for each convolution layer were employed. The ridge regression regularization technique used for both the kernel and bias regularization and the parameters were set to 0.005 and 0.01, respectively. Moreover, the dropout rates for the three convolution layers were set on 0.3 and the dropout of the fully connected layer to 0.5.

Then, the model complied with the Adam optimizer, which is an algorithm for first-order gradient-based optimization of stochastic objective functions based on the adaptive estimates of lower-order moments [43]. Furthermore, the binary cross-entropy loss function was used to measure the performance of our classification model.

One of the essential conditions for achieving excellent performance in a deep neural network is a large amount of training data. Image augmentation is a technique used when the training set is not large enough. It uses some image processing methods, such as random rotations, horizontal/vertical flips or shifts, sheer, changes the image brightness, etc. to artificially increase the amount of data to boost the performance of a deep neural network.

In the current study, 12,000 images were used from each of the three views: Sagittal, coronal and axial coming equally from each group (CN, EMCI and LMCI) for a total of 36,000 images used in our study.

These three mentioned groups were classified based on the three different views, and for each view there were just 4000 images per group. Therefore, this study decided to use image augmentation to generate additional training data by means of sheering, random rotation and zooming. In the following, 70 percent of the images for training and 30 percent for testing were randomly chosen. Further, 20 percent of the training set were selected for the validation set, which provides an unbiased evaluation of the proposed model during the training phase. The batch size was set to 512 images and used 300 epochs for our CNN.

**Figure 3:**
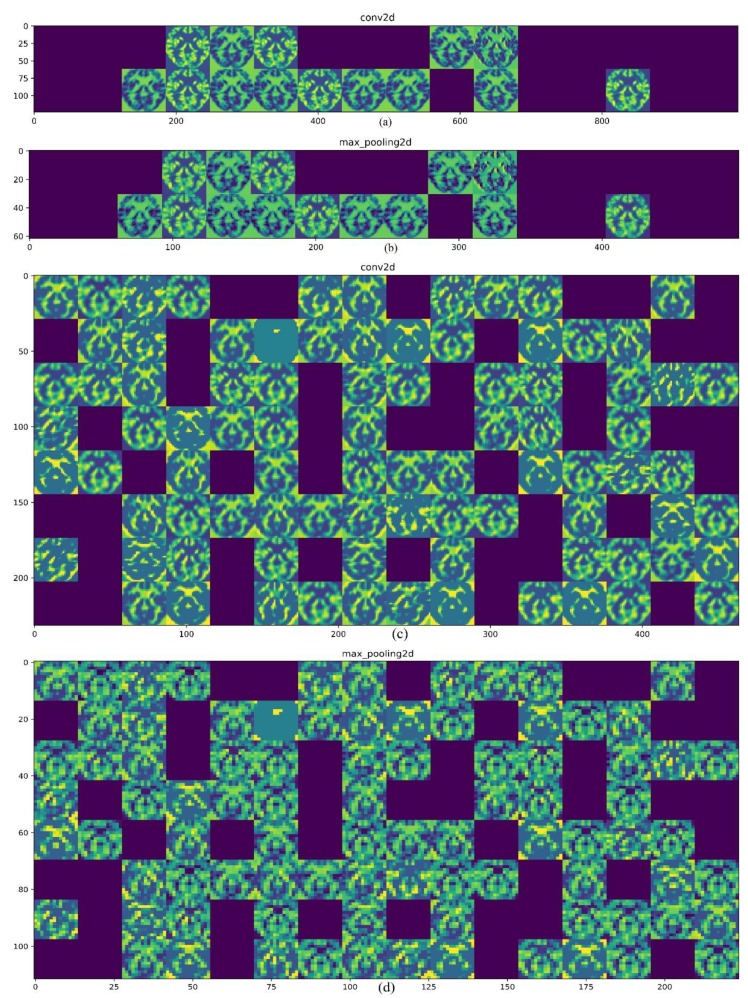
Illustration of the convolutional neural network (CNN) layers output. (**a**) First convolution layer output; (**b**) first max-pooling layer output; (**c**) second convolution layer output; (**d**) second max-pooling layer output.

One of the ambiguities of a CNN is how it processes the data, how it extracts the features, and what the features’ map looks like. To answer these questions, this study illustrated the output of the first and second convolution layers and also the max-pooling layers as shown in Figure 3. The input image is an axial view of the MRI similar to what was showed above. In Figure 3a, the 32 filters from the first convolution layer are presented and one can see the first convolution layer retains the input picture shape and information completely. Moreover, the blank columns show the filters that are not activated. The role of the max-pooling layer for reducing the spatial dimension of the feature maps is presented in Figure 3b. In a CNN, the deeper that is gone, the lesser the output is interpretable, and more image class-related features can be extracted. As shown in Figure 3c, the output of the second convolution layer (128 filters) is less transparent than the first layer. Finally, Figure 3d shows the output of the second map-pooling layer.

The performance of the proposed CAD system was evaluated using five metrics which are accuracy, sensitivity, specificity, F-score, and receiver operating characteristic-area under the curve (AUC-ROC). The parameters definitions are as follows:

(1) $$\mathrm{Sensitivity}=\frac{\mathrm{TP}}{\mathrm{TP}+\mathrm{FN}}\times 100$$

(2) $$\mathrm{Specificity}=\frac{\mathrm{TN}}{\mathrm{TN}+\mathrm{FP}}\times 100$$

(3) $$\mathrm{Accuracy}=\frac{\mathrm{TP}+\mathrm{TN}}{\mathrm{TP}+\mathrm{FP}+\mathrm{TN}+\mathrm{FN}}\times 100$$

(4) $$F-\mathrm{Score}=2\times \frac{P\times R}{P+R}\times 100$$

In this study TP, TN, FP and FN denote the true positive (i.e., the number of EMCI or LMCI patients who were correctly classified), true negative (i.e., the number of NC, which were correctly classified), false positive (i.e., the number of EMCI or LMCI who were classified as NC) and false negative (i.e., the number of NC group, which were classified as EMCI or LMCI) respectively. Further, P and R in the F-score equation are denoted by the precision ($\frac{\mathrm{TP}}{\mathrm{TP}+\mathrm{FP}}$) and recall ($\frac{\mathrm{TP}}{\mathrm{TP}+\mathrm{FN}}$), respectively.

According to the definition above, sensitivity reflects how many EMCI or LMCI subjects were detected accurately. The higher the sensitivity, the fewer AD cases missed. The specificity reflects how many NC cases were detected accurately. The higher the specificity, the fewer normal subjects were misrecognized as EMCI or LMCI. The accuracy represents the ability of the designed system to differentiate between NC, EMCI, and LMCI groups correctly. In addition, the F-score which has a realistic accuracy measurement of the test ROC represents the probability curve, and AUC illustrates the measure of separability. Using these metrics together, the performance of the proposed method can be evaluated comprehensively.

## 4. Results

### 4.1. Classification of CN and LMCI

**Figure 4:**
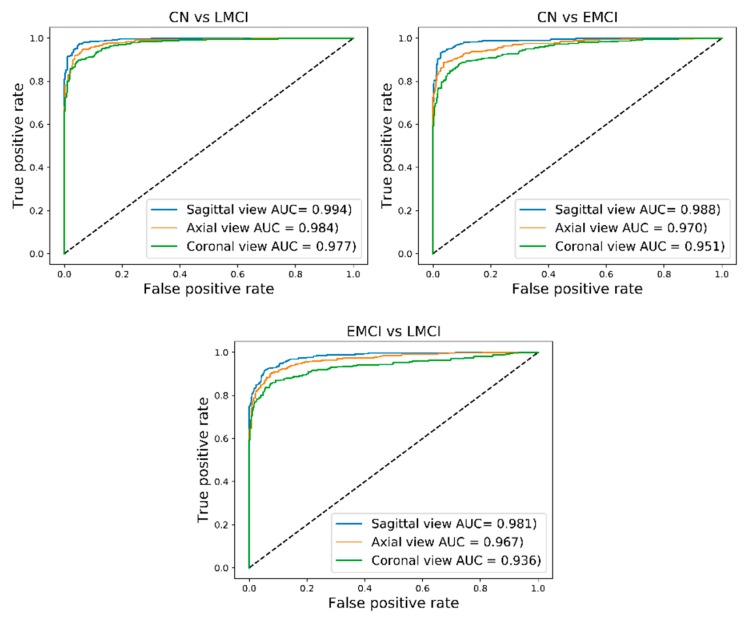
Receiver operating characteristic-area under the curve (ROC-AUC) results of the sagittal, coronal, and axial views.

The classification results for pairs of CN/LMCI for sagittal, coronal, and axial views are shown in Table 2 and also ROC-AUC values are presented in Figure 4. The best classification results were attained from the sagittal view of GM images for the CN and LMCI groups with an accuracy of 94.54%, an F-score of 94.84%, and an AUC of 99.40% (sensitivity of 91.70%, specificity of 97.96). For the axial view, the proposed CAD system reached an accuracy of 93.18%, an F-score of 93.53%, and an AUC of 98.40% (sensitivity of 90.02%, specificity of 97.01%). Moreover, the results for the differentiation between CN/LMCI for the coronal view gave an accuracy of 91.65%, an F-score of 92.19%, and an AUC of 97.70% (sensitivity of 90.28%, specificity of 93.30%).

### 4.2. Classification of CN and EMCI

For the pairs of CN/EMCI, the best classification results were achieved for the sagittal view with an accuracy of 93.96%, an F-score of 94.25%, and an AUC of 98.80% (sensitivity of 90.46%, specificity 98.19%). The views of the axial and coronal obtained lower results than the sagittal view. The achieved results for the axial view were an accuracy of 90.99%, an F-score of 91.65%, an AUC of 97% (sensitivity of 90.63%, specificity of 91.42%). For the coronal view, an accuracy 89.21%, an F-score of 89.96%, and an AUC of 95.10% (sensitivity of 88.60%, specificity 89.95%) was obtained. The classification results and ROC-AUC values are shown in Table 2 and Figure 4.

### 4.3. Classification of EMCI and LMCI

The highest overall classification results for the EMCI and LMCI groups which were obtained for the sagittal view are as follows: Sensitivity of 91.48%, specificity of 94.82%, an accuracy of 93%, an F-score 93.46%, and AUC of 98.10%. The classification results of the axial view reached a sensitivity of 87.01%, a specificity of 94.57%, an accuracy of 90.45%, an F-score of 90.86, and an AUC of 96.70%. The sensitivity, specificity, accuracy, F-score, and AUC of the coronal view were 85.44%, 92.07%, 88.45%, 88.98%, and 93.60%, respectively. The classification results of EMCI and LMCI groups are shown in Table 2, and the ROC-AUC values are illustrated in Figure 4.

## 5. Discussion

Researchers have recently conducted several studies looking into the early diagnosis of AD using deep learning techniques, such as convolutional neural networks (CNNs) [44,45,46]. This research has achieved high accuracy for the classification of CN individuals, the patients with MCI, and AD patients.

MCI status is critical in the early diagnosis of AD because patients with late MCI (LMCI) are classified as having a very high risk of conversion to AD [47], and, more importantly, EMCI can be considered to be the starting point of AD. An accurate and reliable diagnosis of MCI can result in identifying individuals who are at an increased risk of the progression to dementia, and opens doors for providing the potential and routine treatment and gives people the opportunity to plan for the future. Thus, designing an accurate and reliable CAD system for the classification of CN, EMCI, and LMCI patients could be a substantial step forward towards the aging research field. This study, therefore, focused on discriminating CN people from the patients with EMCI and LMCI.

In recent years, deep learning, mainly employing CNNs, has become popular for solving complex tasks, such as image and video processing. This fully trainable system outperforms the traditional methods of classification when using very large data, and unlike conventional machine learning methods, it does not require manual feature extraction steps. A CNN was therefore used in the current study to automatically extract the discriminative features for classifying CN, EMCI, and LMCI patients. The performance of the CAD system the authors have designed has been evaluated using the five parameters of accuracy, F-score, sensitivity, specificity and ROC-AUC.

Several CNN architectures were tried and tested to obtain a reliable and accurate CAD system for the classification of the three groups mentioned above, and the best results were achieved with a CNN consisting of three convolution layers of 32, 128 and 512 filters, respectively. Each convolution layer was also accompanied by a max-pooling layer for down-sampling the spatial dimensions of the input data. It is worth mentioning that to prevent the co-adaptation of feature detectors [48] and to solve the overfitting problem, a dropout was employed for each convolution layer. Finally, the network includes a fully connected layer that is connected to all the neurons in the last convolution layer and a classification layer with a sigmoid activation function that is used to classify the images.

The ADNI MRI data were randomly divided into two groups, one for training and one for testing, comprising 70% and 30% of the total dataset, respectively. The GM was extracted from MRI images of the sagittal, axial and coronal planes and fed into the CNN described above. As shown in Table 2, our proposed deep learning-based CAD system obtained high values for accuracy, F-score, ROC-AUC as well as sensitivity and specificity when attempting to classify the following pairs: CN versus EMCI, CN versus LMCI, and EMCI versus LMCI. The proposed method yielded the best overall results with CN versus LMCI pairs using a sagittal view (accuracy of 94.54%, F-score of 94.84%, and AUC of 99.40%). Moreover, our results show that the sagittal view also leads to better classification performance for CN versus EMCI pairs and EMCI versus LMCI pairs, (accuracy of 93.96%, F-score of 94.25%, AUC of 98.80%, F-score of 93.46%, and an AUC of 98.10%, respectively).

Recently, several studies using different approaches have investigated the early diagnosis of AD based on the classification of healthy normal people, patients with MCI, and AD patients [29,49,50,51]. However, to the best of the authors’ knowledge, there are only three studies that have investigated the classification of healthy normal people, EMCI patients, and LMCI patients [52,53,54]. The highest accuracy achieved by Korolev et al. was 73% for LMCI versus NC, 67% for LMCI versus EMCI, and 63% for EMCI versus NC. Cabrera-León et al. reported an average accuracy for several different methods, of which the best was 57.6%, achieved using the random forest method. The highest F-scores reported by Singh et al. were 83.25% for cognitively unimpaired controls (CU) versus LMCI, 72% for CU versus EMCI, and 68.44% for LMCI versus EMCI.

Thus, comparing this study’s results with the results reported by other studies, it can be concluded that our proposed CAD system is a more reliable and accurate approach.

## 6. Conclusions

Deep neural networks, especially convolutional neural networks, can provide meaningful information for the diagnosis and prognosis of MCI. In this paper, a CNN based method was proposed for extracting discriminative features from structural MRI, with the aim of the diagnosis of EMCI and LMCI and the classification between these two groups and healthy subjects. The proposed method can lead to several benefits for potential MCI individuals and also can lead to an early diagnosis of AD. The experimental results on the ADNI database for 600 subjects demonstrated that our proposed method for feature extraction and classification delivered a high accuracy for the EMCI, LMCI, and CN groups. The best results were achieved for the classification between CN and LMCI groups in the sagittal view and also, the pairs of EMCI/LMCI have achieved slightly better accuracy than CN/EMCI concerning all views of the MRI.

The proposed method yielded a 94.54% classification accuracy (94.84% F-score and 99.40% AUC) for CN versus LMCI, 93.96% classification accuracy for the pairs of CN/EMCI (94.25% F-score and 98.80% AUC), and 93.00% classification accuracy for the classification of the pairs of EMCI/LMCI (93.46% F-score and 98.10% AUC) which all of the above mentioned results achieved from the sagittal view. The above-mentioned results demonstrate higher reliability and precision of our proposed method for the diagnosis of MCI and the classification between the three groups of CN, EMCI, and LMCI.

## Figures and Tables

**Table 1:** The subjects’ clinical and demographic characteristics. For each group, *N* represents the total number of subjects, *M* and *F* show number of males and females, along with the average age, standard deviation (SD) and average mini-mental state examination (MMSE) score.

	CN (*N* = 200; 112 *F*/88 *M*)		EMCI (*N* = 200;93 *F*/107 *M*)		LMCI (*N* = 200; 84 *F*/116 *M*)	
	Mean	SD	Mean	SD	Mean	SD
Age	74.2	6.1	68.2	6.9	71.1	7.2
MMSE	28.8	1.3	28.4	1.2	27.3	1.8

**Table 2:** The classification results of the control normal (CN) versus early mild cognitive impairment (EMCI), CN versus late mild cognitive impairment (LMCI) and EMCI versus LMCI.

	MRI Views	Sensitivity (%)	Specificity (%)	Accuracy (%)	F-Score (%)	AUC (%)
CN vs. LMCI	Sagittal	91.70	97.96	94.54	94.84	99.40
	Axial	90.02	97.01	93.18	93.53	98.40
	Coronal	90.28	93.30	91.65	92.19	97.70
CN vs. EMCI	Sagittal	90.46	98.19	93.96	94.25	98.80
	Axial	90.63	91.42	90.99	91.65	97.00
	Coronal	88.60	89.95	89.21	89.96	95.10
EMCI vs. LMCI	Sagittal	91.48	94.82	93.00	93.46	98.10
	Axial	87.01	94.57	90.45	90.86	96.70
	Coronal	85.44	92.07	88.45	88.98	93.60
